# Predictors of successful discontinuation of antipsychotics and antidepressants

**DOI:** 10.1017/S0033291721005146

**Published:** 2023-05

**Authors:** Tania M. Lincoln, Daniel Sommer, Mariana Quazzola, Tatjana Witzgall, Björn Schlier

**Affiliations:** Universität Hamburg, Hamburg, Germany

**Keywords:** Alliance, depression, mindfulness, schizophrenia, tapering, withdrawal

## Abstract

**Background:**

To offer support for patients who decide to discontinue antipsychotic and antidepressant medication, identifying which potentially modifiable factors correlate with discontinuation success is crucial. Here, we analyzed the predictive value of the professional support received, circumstances prior to discontinuation, a strategy of discontinuation, and use of functional and non-functional coping strategies during discontinuation on self-reported discontinuation success and on objective discontinuation.

**Methods:**

Patients who had attempted discontinuing antipsychotics (AP) and/or antidepressants (AD) during the past 5 years (*n* = 316) completed an online survey including questions on subjective and objective discontinuation success, sociodemographic, clinical and medication-related factors, and scales to assess the putative predictors.

**Results:**

A regression model with all significant predictors explained 20–30% of the variance in discontinuation success for AD and 30–40% for AP. After controlling for baseline sociodemographic, clinical and medication-related factors, the most consistent predictor of subjective discontinuation success was self-care behavior, in particular mindfulness, relaxation and making use of supportive relationships. Other predictors depended on the type of medication: For AD, good alliance with the prescribing physician predicted higher subjective success whereas gradual tapering *per se* was associated with lower subjective success and a lower chance of full discontinuation. In those tapering off AP, leaving time to adjust between dose reductions was associated with higher subjective success and fewer negative effects.

**Conclusions:**

The findings can inform evidence-based clinical guidelines and interventions aiming to support patients during discontinuation. Further studies powered to take interactions between variables into account are needed to improve the prediction of successful discontinuation.

## Introduction

Although detrimental effects of long-term use (⩾ 2 years) of antipsychotics (AP) and antidepressants (AD) have raised critical debates (Maslej et al., 2017; Murray et al., 2016), their long-term prescriptions are continuing to rise (e.g. Mars et al. 2017; Shoham, Cooper, Lewis, Bebbington, & McManus, 2021; Yu, Zhang, Zheng, & Yu, 2020). Some of these prescriptions lack an evidence-base altogether (Mangin et al., 2018; Piek, Kollen, van der Meer, Penninx, & Nolen, 2014), but even for conditions that are assumed to benefit from maintenance therapy beyond the acute phase, i.e. psychosis and chronic, reoccurring depression, the evidence-base for long-term prescriptions is meager. Only about one in four or five patients benefit from maintenance on AP (Leucht et al., 2012) or AD (Geddes et al., 2003; Glue, Donovan, Kolluri, & Emir, 2010) and withdrawal trial designs have been criticized for their short follow-up periods that leave open whether the effects hold up over periods beyond 1 or 2 years (Hengartner, 2020; Moncrieff, 2015). This insufficient state of knowledge has resulted in vague treatment guidelines on maintenance therapy, both for AP and for AD (i.e. ‘at least’ 1 (AD) or 2 (AP) years after a first episode and ‘longer’ in the case of multiple episodes (DGPPN, 2017, 2019; NICE, 2009a, 2009b). This vagueness may have encouraged the costly and potentially harmful clinical practice of long-term medication. Many clinicians' reluctance to support discontinuation is further fuelled by the lack of evidence-based guidelines on safe discontinuation both for AP and for AD.

In order to arrive at evidence-based guidelines for successful discontinuation, we need to understand which factors determine discontinuation success. So far, research has focused largely on *static* sociodemographic and patient characteristics to inform decisions about which subgroup of patients are more likely to benefit from discontinuation. For AP, the overall picture that emerged from a comprehensive review of 37 studies (Tani et al., 2018) is that some of the factors known to predict better long-term outcomes (i.e. older age, shorter duration of untreated psychosis, higher functioning at onset of disorder, and fewer past relapses) also predict discontinuation success. Also, a lower dose and no concomitant medication at the time of discontinuation are likely predictors of discontinuation success. For AD, a review of 13 studies found no conclusive pattern of static predictors of successful discontinuation (Berwian, Walter, Seifritz, & Huys, 2017).

Potentially *modifiable* predictors of discontinuation success that could inform how to guide patients toward successful discontinuation have received less attention. The few existing quantitative studies focused on the preparation of the discontinuation attempt, the method used, or the strategies employed to cope with reoccurring symptoms or withdrawal symptoms (Brandt et al., 2020; Cosci & Chouinard, 2020; Davies et al., 2019; Fava, Gatti, Belaise, Guidi, & Offidani, 2015; Guy, Brown, Lewis, & Horowitz, 2020). Some studies have tested whether gradual or abrupt discontinuation is more effective, with most (Baldessarini, Tondo, Ghiani, & Lepri, 2010; Groot & van Os, 2020; Viguera, 1997) but not all (Batelaan et al., 2017; Khan et al., 2014; Leucht et al., 2012) indicating gradual withdrawal to be advantageous. It has been suggested, both for AP and for AD, that these effects could depend on the tapering plan (Horowitz, Jauhar, Natesan, Murray, & Taylor, 2021; Horowitz & Taylor, 2019). Observational evidence also indicates that the success of gradual tapering could depend on the specific type and class of drug (Framer, 2021), which is unsurprising given the huge variation in the pattern and quantity of withdrawal symptoms they produce (Cosci & Chouinard, 2020). Several case studies indicate that supporting discontinuation with psychological therapy may be beneficial (Belaise, Gatti, Chouinard, & Chouinard, 2014; Bowers et al., 2020; Cromarty, Jonsson, Moorhead, & Freeston, 2011). Other targetable factors likely to impact on discontinuation success of AP and AD can be derived from patient reports of successful strategies (Huijbers, Wentink, Simons, Spijker, & Speckens, 2020; Lehmann, 2013; Ostrow, Jessell, Hurd, Darrow, & Cohen, 2017; Salomon, Hamilton, & Elsom, 2014) and from discontinuation guidelines informed by clinical experience (e.g. Darton, 2016; Deutsche Gesellschaft für Soziale Psychiatrie, 2014; Hall, 2012). These include the personal circumstances prior to discontinuation (i.e. the living and financial situation, relationships, physical health), support by mental health professionals (shared decision making, therapeutic alliance, receiving psychological therapy), functional coping and self-care strategies (e.g. seeking social support, maintaining physical health, staying mindful) and refraining from unhealthy strategies (e.g. substance use). Given the evidence for the relapse-preventive effect of mindfulness interventions in depression (Kuyken et al., 2019) and the promising effects of mindfulness on emotional well-being in psychosis (Vignaud, Reilly, Donde, Haesebaert, & Brunelin, 2019), mindfulness skills, in particular, may help to cope with the emotional turbulence associated with discontinuation of both AP and AD.

In order to predict successful discontinuation, the concept itself also warrants consideration. The classical withdrawal studies largely focused on clinician-rated relapse and rehospitalization as an outcome (Leucht et al., 2012; Maund et al., 2019). However, we have argued that successful discontinuation goes beyond the absence of relapse and should include the subjective feeling of success and the whole range of negative effects (e.g. withdrawal symptoms, affective instability), and positive effects (e.g. increased sense of self-efficacy, increased awareness of feelings). Hence, we have validated a questionnaire to assess these dimensions of successful discontinuation of AP and/or AD (Lincoln, Sommer, Könemund, & Schlier, 2021). Here, we use this scale and the validation sample to identify predictors of successful discontinuation derived from previous research and recommendations from clinical practice. To this aim, we analyzed the predictive value of static and modifiable factors on the objective and subjective success and on the positive and negative effects of discontinuing AP and AD. The static factors included (a) baseline sociodemographic (i.e. age, gender, education) and (b) clinical and medication-related factors (duration of disorder, amount and class of medication being discontinued, dose at time-point of discontinuation, concomitant medication during the discontinuation process). The modifiable factors included (c) the professional support received (shared decision-making, therapeutic alliance, psychological therapy), (d) the circumstances prior to discontinuation, (e) the strategy of discontinuation (preparation & tapering method), and (f) the use of functional (e.g. mindful self-care, making use of social support) and dysfunctional (substance use) coping strategies. Finally, we tested whether the modifiable predictors (c-f) explain variance in discontinuation success over and above the static predictors (a-b) and whether the class of medication (AP *v.* AD) moderates the effect of the predictors on discontinuation success.

## Methods

### Procedure

We conducted an online survey in German-speaking countries, including respondents with a minimum age of 18 and past or current intake of AD or AP. To minimize memory bias while securing ecological validity, participants had to either have started, completed, or continued a discontinuation attempt during the past 5 years. Participants were recruited via an advertisement in online panels and websites with a focus on mental health and via flyers in various facilities (see Lincoln et al., 2021 for further details). The survey took 65 min on average and was programmed using the online-survey platform EFS (QuestBack GmbH, 2017). Written informed consent was obtained followed by assessment of socio-demographic data, clinical and related variables and the questionnaires described below.

### Assessments

#### Discontinuation success

We used the *Discontinuation Success Scale* (DSS) (Lincoln et al., 2021), a 24-item self-report questionnaire to assess the subjective feeling of success (six items), negative (nine items) and positive (nine items) effects of discontinuation. Items are rated on 5-point rating scales (1 = ‘do not agree at all’, 5 = ‘agree completely’). The questionnaire has good construct validity and criterion validity with objective discontinuation and subjective well-being. It has demonstrated sufficient model fit for the participants discontinuing AP or AD, respectively. Cronbachs alpha was sufficient for all three subscales (subjective success: *α* = 0.88, positive effects: *α* = 0.94, negative effects: *α* = 0.91).

Objective discontinuation was assessed by asking participants whether they had stopped taking the medication they had intended to discontinue, or were taking the medication in a lower dose, the same dose, or a higher dose than before. To secure sufficient statistical power for the prediction of objective discontinuation, this variable was dichotomized for the analyses (1 = stopped taking the medication the participant had planned to discontinue) *v.* 0 ( = taking the medication in a lower dose, the same dose, or a higher dose than before).

#### Clinical and medication history

We assessed (a) the duration of the disorder, (b) the duration of medication intake, (c) the type of medication discontinued clustered by groups (AD: SSRI, SNRI, tricyclic/tetracyclic; AP: first-generation/second generation), (d) whether one or multiple drugs were being discontinued, and (e) the presence or absence of concomitant medication while discontinuing the target medication.

#### Professional support

To assess shared decision-making with the prescribing physician regarding discontinuation, we used the nine-item version of the German questionnaire *Fragebogen zur Partizipativen Entscheidungsfindung* (PEF-FB-9) [shared decision-making questionnaire] (Scholl, Kriston, & Härter, 2011), which has been shown to have a high internal consistency of *α* = 0.94 (Scholl et al., 2011). For this study, we adapted the scale to refer to the topic of discontinuation.

To assess the quality of the relationship with the prescribing clinician, we used the validated German version (Bassler, Potratz, & Krauthauser, 1995) of the six-item subscale *therapeutic relationship* of the *Helping Alliance Questionnaire* (HAQ) (Luborsky et al., 1996). High internal consistency of *α* = 0.89 has been reported for this subscale (Bassler et al., 1995). We phrased the items in the past tense, changed the word ‘therapist’ to ‘doctor’, and asked participants to refer their responses to the prescribing physician.

Finally, participants indicated whether they had received psychological therapy during the discontinuation attempt (yes/no-question).

#### Circumstances prior to discontinuation

To assess stability in key areas of life, we used the seven-item subscale *stress due to instability* from the German *Stress- and Coping- Inventory* (SCI) (Satow, 2012) that assesses the amount of instability regarding finances, home, partnership, job, health, family and friends with sufficient internal consistency (*α* = 0.72) (Satow, 2012). For our purpose, we changed the instruction to: ‘How stressed did you feel by following uncertainties in the last few weeks before your discontinuation attempt?’.

#### Discontinuation strategy

The strategy of discontinuation was assessed with a self-developed two-part questionnaire. The first part assessed whether the participant had sought information and prepared the discontinuation attempt (*preparation*, three items, e.g. ‘I made preparations before starting to discontinue’, see Supplement 1, Table S1), and had followed a *tapering plan* (three items, e.g. ‘I followed a detailed plan on how to reduce my medication’, see Supplement 1, Table S1). Participants indicating to have followed a tapering plan (*n* = 247) were asked whether their plan included *adjustment phases* following each reduction step (two items, e.g. ‘I only continued discontinuation when I felt stable enough with the current dosage’, see Supplement 1 Table S2) and whether it allowed for a *flexible change* of the dose (two items, e.g. ‘I followed my gut feeling when discontinuing’, see Supplement 1, Table S2). Furthermore, participants described their *pacing* by selecting one out of eight different types of recommended tapering strategies (Deutsche Gesellschaft für Soziale Psychiatrie, 2014; Hall, 2012), ranging from slow (<10% reduction of the most recent dose every 4–6 weeks) to fast (>50% reduction or more of the most recent dose every 2–4 weeks).

For the items assessing *preparation* and *tapering,* principal component analysis (PCA) and subsequent exploratory factor analysis (EFA) supported the two-factor-model, with sufficient model fit in confirmatory factor analysis (CFA): *CFI* = 0.991, *RMSEA* = 0.060, *SRMR* = 0.030, loadings: 0.634–0.952. For the items *adjustment phases* and *flexible change*, PCA yielded two components with eigenvalues above 1. CFA indicated a pattern of largely standardized loadings (loadings: 0.318–0.950; see Supplement 1 for details on both factor analyses). The variable *pacing* was dichotomized to 0 = ‘slow’ (<10% reduction every 4–6 weeks, *n* = 48) and 1 = ‘medium or fast’ (>10% reduction every 4–6 weeks, *n* = 144).

#### Functional and dysfunctional coping strategies

We assessed self-care strategies with the *Mindful Self-Care Scale* (MSCS) (Cook-Cottone & Guyker, 2018). The MSCS addresses six domains of self-care: *physical care* (eight items), *supportive relationships* (five items), *mindful awareness* (four items), *self-compassion and purpose* (six items), *mindful relaxation* (six items), and *supportive structure* (four items, see Supplement 1, Table S3 for a full item list). Items are answered on a five-point rating scale (1 = ‘never’, 5 = ‘almost always’). A higher score indicates more self-care behavior. The German version was developed by translation and blind back-translation. The participants answered the questions in reference to the period of the discontinuation attempt. Due to the translation and adaption, we re-assessed the MSCS factor structure and revised the item selection where necessary. Parallel analysis and optimal coordinates of the scree plot (Raîche, Walls, Magis, Riopel, & Blais, 2013) indicated six factors. The loadings of a promax-rotated EFA roughly mirrored the original subscales. Three items for physical self-care showed low overall loadings (drinking enough water, planned meals and drinks, practicing yoga/mind-body exercises; all loadings ⩽0.26) and were removed. One relaxation-item (‘I did something interpersonal to relax’) showed a higher loading on supportive relationships and the two purpose items from the self-compassion/purpose subscale (‘I experienced meaning and/or a larger purpose in my work/school life’ and ‘I experienced meaning and/or a larger purpose in my private/personal life’) showed higher loadings on the supportive structure factor (all Δ_loadings_ > 0.30), so these three items were allocated to new factors. CFA of the revised six-factor model yielded acceptable model fit (CFI = 0.909, RMSEA = 0.056, SRMR = 0.063, see Supplement 1 for details) and a substantially better fit than the original model (CFI = 0.834, RMSEA = 0.070, SRMR = 0.081). Internal consistencies were sufficient (0.75 ⩽ *α* ⩽ 0.89) for all scales except relaxation (*α* = 0.63).

Substance use was assessed with four items asking about the frequency of the consumption of alcohol, nicotine, cannabis, and other psychoactive substances respectively on a scale ranging from 1 to 7 (1 = ‘never’, 7 = ‘every day’). PCA indicated a unifactorial model, which was found to fit sufficiently in CFA (CFI = 0.972, RMSEA = 0.062, SRMR = 0.029, loadings: 0.374–0.638), albeit with low internal consistency (*α* = 0.41). For this study, we used the total score.

### Data analysis

We conducted the data analysis using R 3.4.2. To explore which putative predictor had an impact on discontinuation success, we calculated single-predictor linear (subjective success) and logistic (objective discontinuation) regression models. Regression analyses were calculated separately for those aiming to discontinue at least one AD and those aiming to discontinue at least one AP with each of the discontinuation success variables (subjective success, positive effects, negative effects, objective discontinuation) as a dependent variable and one predictor as an independent variable. Following this, all significant predictors were entered into multiple regression models, with significant *static factors* – demographic variables (age, gender, education), and clinical and medical history (duration of disorder, type of medication, duration of intake, one *v.* multiple drugs discontinued, concomitant medication) – entered in a first step. Psychosocial predictors (professional support, circumstances, discontinuation strategy, coping strategies) were entered in a second step to illustrate the additional variance (ΔR^2^) of *modifiable factors* and the overall explained variance in discontinuation success. Furthermore, the most influential psychosocial predictors for each of the discontinuation variables were identified with these models.

We then used the full sample to test for interaction effects of AD *v.* AP. An interaction effect of the respective predictor and AP-discontinuation (discontinuation intent includes AP; yes *v.* no) was entered to all single predictor models that previously yielded a significant main effect in the AD and/or AP single-predictor regression models.

All analyses were performed with the data as answered by the participants without replacing missing values. Missing values ranged between 0.00% and 1.90% per variable, except for drug consumption (5.06%).

## Results

### Participants

A sample of 316 participants (of 396 who answered the first part of the survey concerning the validation of the DSS) completed the full survey and were included in the analysis (73.4% female; age: *M* = 38.9 years, s.d. *=* *12.4*, range = 18–72). Two-thirds had a university degree (41.5%) or a university entrance diploma (28.8%). Close to half reported being employed/self-employed (48.7%), 19.6% were retired (early retirement: 17.4%; pensioner: 2.2%), and 15.2% were enrolled in school, university or vocational training.

On average, participants reported to have 2.10 diagnoses (s.d. = 1.12, range = 0–7), with two participants not knowing their diagnoses. Most frequent diagnoses were depressive disorders (71.2%), anxiety disorders (43.7%), personality disorders (22.5%), PTSD (20.9%), psychotic disorders (15.5%), eating disorders (9.2%) and bipolar disorders (6.0%). The average duration of the disorder was 11.65 years (s.d. = 9.75)

The majority (*n* = 237; 75.0%) attempted to discontinue one drug, a quarter of the sample discontinued multiple drugs. The discontinued medication included one or more AD for 82.9% (*n* = 262) and one or more AP for 29.7% (*n* = 94), with 12.3% (*n* = 39) attempting to discontinue a combination of AD and AP. Among ADs, 129 (49.2%) discontinuation attempts involved SSRI, 104 (39.8%) involved SNRI, and 46 (17.6%) involved tricyclic or tetracyclic AD. Among APs, 84 (89.4%) discontinuation attempts involved one or more second-generation AP (including one participant who discontinued Clozapine) and 26 (27.7%) involved discontinuation of one or more first-generation AP. Participants were taking medication for an average of 9.70 years (s.d. = 7.74) before discontinuation.

About half of the sample (48.7%) received psychological therapy during the time of their discontinuation attempt. A minority of the participants (*n* = 55, 17.4%) continued taking other medication during their discontinuation attempt.

At the time of the survey, 54.4% of the participants had stopped taking medication as planned (AD: 53.1%; AP: 55.3%), 33.2% had managed to reduce their dose (AD: 35.1%; AP: 36.1%), 10.4% were still taking the same (AD: 9.9%; AP: 7.4%), and 1.6% a higher dose (AD: 1.5%; AP: 1.1%). On average, participants reported to have started their discontinuation attempt 1.80 years ago (s.d. = 2.69, range = 0.01–10, excluding one outlier who reported to have started it 38 years ago). Overall, 38.9% of the participants reported that their discontinuation attempt was still ongoing.

Those who had stopped taking the medication as planned reported to have been off the specified medication for *M* = 1.48 years (s.d. = 1.53; range = 0.02–5.5), with 88 participants being off medication for <2 years and 39 for ⩾2 years.

### AD discontinuation success

Table 1 shows the results of all AD single predictor regression models. The most consistent associations were found for self-care behavior, in particular for *mindful awareness*, *self-compassion*, *supportive relationships* and *supportive structures*. *Shared decision-making* and *therapeutic alliance* predicted more subjective success and fewer negative effects. However, these effects were limited to the DSS scales (i.e. they were not observed for objective discontinuation). Unstable circumstances, in contrast, were associated with objective discontinuation, but not with subjective success.

**Table 1.** Single predictor regression analyses for all putative psychosocial predictors in the antidepressant discontinuation group

Predictor	DSS success			DSS positive effects			DSS negative effects			Objective discontinuation	
	*b*	*t*	*R*^2^	*b*	*t*	*R*^2^	*b*	*t*	*R*^2^	OR	*z*
Sociodemographic data											
Age	0.00	0.13	<0.001	0.03	0.59	0.001	−0.07	−1.52	0.008	0.99	−0.83
Gender (female *v*. male)	−0.46	−0.63	0.002	−0.74	−0.51	0.001	−0.42	−0.32	<0.001	0.43**	**−2.84**
Education (high *v*. low)	−0.36	−0.52	0.001	0.93	0.68	0.002	−2.16^+^	−1.72	0.011	1.19	0.66
Clinical and medication history											
Duration of disorder	−0.01	−0.41	0.001	−0.02	−0.36	0.001	0.00	0.08	<0.001	1.02	−0.90
Discontinuation of SSRI	0.42	0.67	0.002	−0.92	−0.73	0.002	−0.10	−0.08	<0.001	1.18	0.07
Discontinuation of SNRI	−1.14^+^	−1.80	0.012	−1.01	−0.78	0.002	0.33	0.28	<0.001	0.79	−0.92
Discontinuation of tri/tetracyclic AD	2.11*	**2.59**	**0.026**	2.55	1.54	0.009	1.39	0.90	0.003	3.08**	**3.10**
Duration of medication intake	0.03	0.65	0.002	0.07	0.86	0.003	−0.01	−0.12	<0.001	0.98	−1.17
Multiple discontinued drugs (*v*. one)	2.37***	**3.38**	**0.042**	3.88**	**2.71**	**0.028**	−0.79	−0.59	0.001	1.37	1.11
Concomitant medication	−0.36	−0.42	0.001	−4.01*	**2.36**	**0.022**	1.44	0.91	0.003	0.61	−1.44
Professional support											
Shared decision-making	0.11***	**3.74**	**0.051**	0.03	0.59	0.001	−0.12*	**−2.24**	**0.019**	0.99	−0.60
Therapeutic alliance	0.75***	**4.20**	**0.065**	0.17	0.45	0.001	−0.99**	**−2.95**	**0.033**	0.91	−1.35
Psychotherapy during attempt	0.22	0.35	<0.001	−0.70	−0.55	0.001	−0.67	−0.58	0.001	0.97	−0.14
Circumstances											
Unstable situation	0.02	0.53	0.001	0.014	0.66	0.002	0.04	0.66	0.002	1.03*	**2.15**
Discontinuation strategy											
Preparation	0.02	0.05	<0.001	0.45	0.64	0.002	−1.15^+^	−1.79	0.012	0.77^+^	−1.89
Followed a tapering plan	−0.06	−0.21	<0.001	−0.31	−0.55	0.001	−0.27	−0.52	0.001	0.67***	**−3.38**
Adjustment phases^a^	0.48^+^	1.71	0.014	0.72	1.24	0.008	−1.27**	**−2.39**	**0.028**	0.71**	**−2.99**
Flexible reduction^a^	−0.47	−1.49	0.011	0.09	0.13	<0.001	0.56	0.92	0.004	1.20	1.44
Accelerated pacing (*v*. slow)^a^	2.61**	**2.80**	**0.049**	−1.05*^+^*	−0.55	0.002	−0.37	−0.24	0.051	2.40*	**2.17**
Coping strategies											
Physical care	−0.49	−1.33	0.007	1.37^+^	1.86	0.013	−0.45	−0.66	0.002	0.96	−0.27
Relaxation	−0.08	0.17	<0.001	3.08***	**3.67**	**0.051**	−0.07	−0.09	<0.001	0.95	−0.27
Mindful awareness	1.50***	**4.72**	**0.080**	3.44***	**5.41**	**0.102**	−3.76***	**−6.54**	0.145	0.98	−0.14
Self-compassion	0.84*	**2.52**	**0.024**	3.40***	**5.32**	**0.102**	−2.62***	**−4.33**	0.069	1.12	0.85
Supportive relationships	0.94**	**2.79**	**0.030**	2.73***	**4.14**	**0.064**	−2.98***	**−4.92**	**0.087**	0.78^+^	−1.82
Supportive structures	0.53	1.62	0.010	3.25***	**4.78**	**0.084**	−2.79***	**−4.39**	**0.071**	0.71*	**−2.40**
Drug consumption	0.53	1.51	0.009	0.62	0.86	0.003	0.09	0.14	<0.001	1.26	1.62

*Note*: Regression analyses with one predictor and one dependent variable.

a Only participants who indicated to have followed a tapering plan responded to the questions on the details of the tapering plan (*n* = 200).

Significance levels: +*p* < 0.1, *p < 0.05, **p < 0.01, ***p < 0.001. Significant effects are printed in bold.

Not following a tapering plan was associated with an increased chance of objective discontinuation. For those who tapered, leaving *adjustment phases* predicted less negative effects but a reduced chance of objective discontinuation, whereas *medium to fast-paced tapering* was associated with more subjective success and an increased chance of objective discontinuation.

Among the *static* sociodemographic, clinical, and medication-related variables, discontinuation of tricyclic/tetracyclic AD was associated with higher subjective success and a higher likelihood of objective discontinuation. Coming off multiple drugs at once was associated with higher subjective reports of success and more positive effects. Female gender was associated with a lower likelihood of objective discontinuation.

A multiple regression model with all significant predictors explained 25.23% of the variance in subjective discontinuation success (*F*(8,141) = 5.95, *p* < 0.001), which was significantly more than the step 1 model using significant sociodemographic, clinical and medication-related variables (Δ*R^2^* = 0.194, *F*(6,141) = 6.10, *p* < 0.001). *Therapeutic alliance* (*b* = 0.82, *T* = 2.89, *p* = 0.004) remained the only significant predictor in Step 2.

For positive effects of discontinuation, 19.98% of the variance (*F*(7,244) = 8.70, *p* < 0.001; improvement from step 1: Δ*R^2^* = 0.153, *F*(5,244) = 9.35, *p* < 0.001) could be explained, but only *mindful awareness* remained a significant predictor in Step 2 (*b* = 1.73, *T* = 2.15, *p* = 0.032).

For negative effects of discontinuation, 24.87% of the variance (*F*(7,187) = 7.87, *p* < 0.001) could be explained (no significant sociodemographic, clinical, medication-related variables to enter in step 1), with *mindful awareness* (*b* = −2.92, *T* = −3.24, *p* < 0.001) remaining significant.

Multiple logistic regression of objective success (*pseudo-R^2^* = 0.224) yielded *adjustment phases* as the only remaining predictor (*OR* = 0.68, *Z* = −2.19, *p* = 0.028) with significant improvement from step 1 (*Δpseudo-R^2^* = 0.095, *χ^2^*(5) = 12.35, *p* = 0.030).

### AP discontinuation success

For the AP single predictor regression models (Table 2), we also found significant effects predominantly for self-care behavior, which did not extend to objective discontinuation. In terms of discontinuation strategy, we found significant effects for *preparation*, *adjustment phases,* and *flexible reduction on various indicators of discontinuation success*. Here too, unstable circumstances were associated with objective discontinuation, but they were also associated with more reported negative effects. Therapeutic alliance and receiving psychotherapy were not predictive of successful AP discontinuation, with single associations even pointing in the opposite direction.

**Table 2.** Single predictor regression analyses for all putative psychosocial predictors in the antipsychotic discontinuation group

Predictor	DSS success			DSS positive effects			DSS negative effects			Objective discontinuation	
	*b*	*t*	*R*^2^	*b*	*t*	*R*^2^	*b*	*t*	*R*^2^	OR	*z*
Sociodemographic data											
Age	0.01	0.13	<0.001	0.02	0.24	0.001	−0.09	−1.08	0.012	0.99	−0.69
Gender (female *v*. male)	−0.16	−0.14	<0.001	−3.46	−1.59	0.027	0.07	0.03	<0.001	0.31*	**−2.33**
Education (high *v*. low)	0.02	0.02	<0.001	−0.65	−0.30	0.001	−2.45	−1.25	0.017	0.76	0.62
Clinical and medication history											
Duration of disorder	−0.01	−0.17	<0.001	0.12	0.90	0.008	−0.02	−0.13	<0.001	1.00	0.14
Discontinuation of 1^st^ gen AP	−1.03	−0.93	0.009	−2.38	−1.06	0.012	4.05*	**2.01**	**0.042**	0.74	−0.64
Discontinuation of 2^nd^ gen AP	1.14	0.74	0.006	6.74*	**2.19**	**0.049**	−6.95*	**−2.51**	**0.064**	2.40	1.32
Duration of medication intake	0.06	0.85	0.008	0.16	1.15	0.014	−0.05	−0.44	0.002	0.99	−0.26
Multiple discontinued drugs (*v*. one)	−0.32	−0.32	0.001	−1.05	−0.51	0.003	4.33*	**2.41**	**0.060**	0.57	−1.33
Concomitant medication	0.98	0.81	0.007	−0.92	−0.37	0.001	0.93	0.42	0.002	0.76	0.54
Professional support											
Shared decision-making	0.06	1.43	0.022	−0.43	−0.75	0.006	−0.06	−0.75	0.006	0.98	−1.39
Therapeutic alliance	0.18	0.64	0.004	−0.42	−0.75	0.006	−0.06	0.13	<0.001	0.69**	**−0.37**
Psychotherapy during attempt	−0.30	−0.30	0.001	−4.15*	**−2.07**	**0.045**	3.31^+^	1.81	0.034	0.79	−0.55
Circumstances											
Unstable situation	−0.01	−0.28	0.001	−0.16	−1.53	0.024	0.27**	**2.92**	**0.085**	1.05*	**1.97**
Discontinuation strategy											
Preparation	1.12*	**2.17**	**0.049**	1.32	1.23	0.016	−2.14*	**2.25**	**0.052**	0.71	−1.49
Followed a tapering plan	0.48	1.07	0.012	−0.58	−0.63	0.004	−0.50	−0.59	0.004	0.74	−1.50
Adjustment phases^a^	1.20**	**2.84**	**0.098**	2.44**	**2.79**	**0.094**	−3.02***	**3.98**	**0.174**	1.02	0.10
Flexible reduction^a^	−0.82^+^	−1.80	0.042	1.59^+^	1.69	0.037	0.85	0.99	0.013	1.54*	**2.03**
Accelerated pacing (*v*. slow)^a^	−1.20	−0.89	0.013	−4.56	−1.56	0.039	1.47	0.57	0.005	0.51	−1.07
Coping strategies											
Physical care	−1.30*	**−2.43**	**0.061**	1.18	1.04	0.012	0.98	0.95	0.010	0.81	−0.91
Relaxation	0.40	0.61	0.004	4.99***	**3.91**	**0.145**	2.17^+^	−1.75	0.033	1.10	0.33
Mindful awareness	1.73***	**3.96**	**0.150**	3.88***	**4.27**	**0.168**	−3.25***	**−3.85**	**0.141**	0.89	−0.59
Self-compassion	0.23	0.44	0.002	3.45**	**3.36**	**0.112**	−1.91^+^	−1.96	0.041	0.98	−0.07
Supportive relationships	1.38*	**2.60**	**0.071**	3.74***	**3.55**	**0.123**	−4.22***	**−4.57**	**0.188**	0.77	−1.13
Supportive structures	0.88	1.61	0.028	4.41***	**4.15**	**0.161**	−3.58***	**3.62**	**0.127**	0.84	−0.71
Drug consumption	−0.50	1.16	0.015	0.58	0.64	0.005	0.98	1.19	0.016	1.52^+^	1.90

*Note*: Regression analyses with one predictor and one dependent variable.

a Only participants who indicated to have followed a tapering plan responded to the questions on the details of the tapering plan (*n* = 76).

+*p* < 0.1, *p < 0.05, **p < 0.01, ***p < 0.001. Significant effects are printed in bold.

Among the *static* variables, discontinuing 2^nd^ generation AP was associated with more subjective success and fewer negative effects. In contrast to AD, coming off multiple drugs at once was associated with more negative effects. Again, female gender was associated with a lower likelihood of objective discontinuation.

A multiple regression model with all significant predictors explained 29.90% of the variance in subjective discontinuation success (*F*(5,70) = 5.97, *p* < 0.001 – no significant static factors to enter in step 1), with *adjustment phases* remaining as a positive (*b* = 0.94, *T* = 2.26, *p* = 0.027) and *physical self-care* as a negative significant predictor in Step 2 (*b* = −1.48, *T* = 2.89, *p* = 0.009).

For positive effects of discontinuation, 36.85% of the variance (*F*(8,68) = 8.70, *p* < 0.001; improvement from step 1: Δ*R^2^* = 0.319, *F*(7,68) = 4.90, *p* < 0.001) could be explained, with *relaxation* (*b* = 4.83, *T* = 2.77, *p* = 0.007) and *mindful awareness* (*b* = 2.97, *T* = 2.16, *p* = 0.034) remaining significant in Step 2.

For negative effects of discontinuation, 40.13% (*F*(8,68) = 5.70, *p* < 0.001; improvement from step 1: Δ*R^2^* = 0.298, *F*(6,68) = 5.63, *p* < 0.001) of the variance could be explained, with *adjustment phases* (*b* = −1.93, *T* = −2.64, *p* = 0.010) and *supportive relationships* (*b* = −2.48, *T* = −2.12, *p* = 0.038) remaining significant in Step 2.

Regarding objective success (*pseudo-R^2^* = 0.232), overall model-fit significantly improved from step 1 to step 2 (*Δpseudo-R^2^* = 0.171, *χ^2^*(3) = 11.11, *p* = 0.011), but no significant predictor remained in Step 2.

### Differential effects for antidepressant *v.* antipsychotic discontinuation

We found six significant interaction effects (see Table 3). Most of these pertained to discontinuation strategy, including the AP-specificity of preparation, adjustment phases being associated with less negative effects in AP *v.* AD, and the AD-specificity of accelerated pacing (on subjective success and objective discontinuation). Additionally, we verified the AP-specificity of the effect of unstable circumstances on negative effects and of therapeutic alliance on objective success.

**Table 3.** Overview of the significant interaction effects in moderation analyses

Outcome	Predictor	Main effect AD	Main effect AP	Interaction effect		
		*b*/OR	*b*/OR	*b*/OR	*T*/*Z*	*p*
DSS subjective success	Preparation	*b* = 0.02	*b* = 1.12*	*b* = 1.42*	2.19	0.029
	Accelerated pacing^a^	*b* = 2.61**	*b* = −1.20	*b* = −3.45*	2.00	0.047
DSS negative effects	Adjustment phases^a^	*b* = −1.27**	*b* = −3.02***	*b* = −2.31*	−2.28	0.024
	Unstable circumstances	*b* = 0.04	*b* = 0.27**	*b* = 0.27*	2.46	0.014
Objective discontinuation	Therapeutic alliance	OR = 0.91	OR = 0.69**	OR = 0.73*	−2.10	0.036
	Accelerated pacing^a^	OR = 2.40*	OR = 0.51	OR = 0.16*	−2.43	0.015

DSS, Discontinuation Success Scale.

*Note*: Regression analyses with one predictor and moderator AP-discontinuation (yes v. no) predicting one dependent variable.

a Only participants who indicated to have followed a tapering plan responded to the questions on the details of the tapering plan.

+*p* < 0.1, *p < 0.05, **p < 0.01, ***p < 0.001.

### Supplementary analyses

To rule out that the results for the dichotomized variable objective discontinuation (complete discontinuation *v.* reduction/same dose/higher dose) were affected by participants who only recently started their discontinuation attempt (and could therefore be on track toward full discontinuation), we recalculated the logistic regression on a subsample excluding those who reported to be on an ongoing discontinuation attempt that had started within the last year. In these analyses, all significant effects remained stable for AD discontinuation (see Supplement 2, Table S2-1). For AP discontinuation (see Supplement 2, Table S2-2), several predictors lost significance, which was likely due to a lack of power, but the overall direction of the associations remained the same.

## Discussion

Aiming to determine factors that predict successful discontinuation in order to inform guidelines, we tested the predictive value of a large set of potentially relevant factors. The percentage of explained variance in the different indicators of discontinuation success ranged from 30% to 40% for AP and from 20% to 30% for AD, which indicates that we managed to assess a significant part of the relevant factors. The findings were consistent in showing that the hypothesized *modifiable* predictors added significantly to the predictions, attesting to their relevance over and above clinical and medication-related *static* predictors.

Before discussing the central findings and their implications, we would like to point to two aspects relevant to the interpretation of the findings: First, the cross-sectional design with retrospective assessment precludes from verifying any of the factors as predictors in a temporal sense. With varying degrees of plausibility, each significant association could also be explained by the reverse causal direction. For example, a person with extreme withdrawal problems might find it difficult to remain mindful of thoughts and feelings. Bearing this in mind, we will nevertheless use the term predictors to present a consistent theoretical framework. Second, although we tested for interactions of medication class (AD *v.* AP), numerous other interaction effects are likely to be relevant. For example, people who took their medication longer may benefit more from a slow tapering strategy. Testing all possible interactions would require a significantly larger sample size, but possible interaction effects need to be kept in mind when interpreting some of the findings.

Self-care behavior, in particular mindfulness, relaxation and supportive relationships were the most consistent predictors of discontinuation success of both AD and AP. In the controlled analyses, mindful awareness predicted more positive effects in people coming off AD and less negative effects for both subsamples. However, mindful awareness did not predict objective discontinuation, indicating that it exerts its effect on coping with the discontinuation process rather than on the discontinuation as such. This makes intuitive sense: Withdrawal symptoms can include a range of symptoms (Brandt et al., 2020; Cosci & Chouinard, 2020; Davies et al., 2019; Fava et al., 2015; Guy et al., 2020) that can be misidentified as a sign of impending relapse. These are likely to trigger catastrophic beliefs (e.g. ‘I am about to relapse’, ‘Relapse would be terrible’ etc.) that accelerate anxious arousal and thereby render relapse more likely (Gumley & Schwannauer, 2006). Mindfulness involves a calm awareness of thoughts, feelings and the body, to enable a conscious selection of the thoughts and feelings to be guided by. Consequently, an ongoing study is testing the effect of mindfulness-based cognitive therapy combined with supported discontinuation of AD compared to supported discontinuation alone (Wentink et al., 2019). Our findings indicate that this approach could also work in the context of AP withdrawal. However, it could be promising to enhance it with relaxation techniques that were predictive of positive discontinuation effects. It could also help to support patients to activate interpersonal supportive resources as these were predictive of fewer negative effects.

The discontinuation strategy was also relevant to success, but this effect was less straightforward. Although a gradual strategy is generally recommended (Horowitz et al., 2021; Horowitz & Taylor, 2019; NICE, 2009a, 2009b), we did not find that a gradual approach *per se* (*v.* abrupt) predicted discontinuation success. In fact - even after controlling for other variables – we found that those who reduced AD all at once were more likely to achieve full discontinuation. Among those who worked with a tapering plan to discontinue AD, leaving time between steps reduced the negative effects but rendered it less likely to reach full discontinuation. In contrast, faster tapering increased the chance of full discontinuation, which is also reflected in the higher self-reported success scores. Given that this finding does not align with clinical observations and previous research that indicates gradual withdrawal to be advantageous (Baldessarini et al., 2010; Groot & van Os, 2020) the limitations of the non-randomized design and the putative interaction effects noted above require particular attention. For instance, it is possible that the effect is driven by a group of patients with milder psychopathology or a short duration of medication intake. This calls for more studies on moderating effects. Nevertheless, the findings question the notion that gradual withdrawal is better *per se*. The overall pattern of findings also indicates that what may constitute a successful tapering strategy for AD depends on whether more value is placed on being off medication quickly or on preventing negative effects. It could thus be helpful to discuss these differential effects with patients in the context of a shared decision-making process.

For AP, leaving time to adjust was significantly associated with subjective success and fewer negative effects in the controlled analyses. In the univariate analyses, good preparation also predicted subjective success. Objective discontinuation was predicted by a ‘flexible reduction strategy’, but this did not hold up in the controlled analysis. Thus, the most promising route to successful discontinuation of AP seems to be leaving time to adjust, which aligns both with expert recommendations (Horowitz et al., 2021) and with reported experiences from peer-supported clinical practice that indicate that ‘adjusting the taper to the individual’ is crucial to success (Framer, 2021).

Encouragingly, shared decision-making and good therapeutic alliance with the prescribing physician were significant predictors of more subjective success and fewer negative effects for AD discontinuation. This points to room for improvement in clinical practice, which is often characterized by an absence of consultation with the prescribing physician (Salomon et al., 2014; Samples & Mojtabai, 2015) or consultation that is not perceived as helpful (Ostrow et al., 2017). Seeing a psychologist does not seem to compensate for a poor alliance with the physician as psychological therapy during the discontinuation period did not predict discontinuation success. The only significant antipsychotic-specific effect even pointed in the opposite direction. This may be due to uncertainties on the sides of clinical psychologists. However, there is work in progress in this domain with informed guidance on how psychologists can use their role to empower patients during the withdrawal process currently being rolled out (Guy, Frederick, Davies, Kolubinski, & Montagu, 2019). Also, researchers are focusing on the potential of psychologically informed specialized internet programs and telephone counseling (Kendrick, 2020).

Other noteworthy findings pertained to medication type: It seems easier to withdraw tricyclic AD than other types, possibly due to the lower quantity of (cognitive) withdrawal symptoms associated with tricyclic AD (Cosci & Chouinard, 2020). Moreover, discontinuation of second-generation AP (*v.* first-generation AP) was associated with more positive and less negative effects. However, the subsample discontinuing first-generation AP was small and previous research provides limited support for the claim of less withdrawal problems with second-generation AP (Cosci & Chouinard, 2020), necessitating replication before further interpreting this finding.

### Strengths and limitations

We limited the time frame to reduce retrospective memory bias, but ideally, we would want to assess predictor variable and success concurrently over the discontinuation period and then use time-lagged analyses to test for significant predictors. This would also have the advantage of being able to capture the full-time period of the discontinuation process for each participant. The time periods since finishing discontinuation varied between participants, however, controlling for this in the analyses did not affect the results. Grouping those who had reduced with those who had remained on the same or moved to a higher dose is debatable as a dose reduction could reflect the first step toward full discontinuation. However, excluding those with recent, uncompleted attempts did not affect the pattern of findings. Also, this was a sample that had set out with the explicit aim to fully stop, and not merely reduce, medication. Another limitation is that the sample is slightly biased toward the more educated. It also includes more women, which is likely due to the fact that they are more likely to receive AD. Also, given that the recruitment took place via online forums, it is likely to be biased toward those struggling with discontinuation. A strength is the samples' heterogeneity in terms of diagnoses and the use of a validated tool to assess discontinuation success.

### Conclusion and outlook

The present study made an important step toward identifying modifiable predictors to inform clinical guidelines on discontinuation of AD and AP. Its findings underline the relevance of the modifiable predictors over and above ‘static predictors’ that have been the focus of research so far. To improve on the subjective success of discontinuation (along with the perception of positive and negative effects), the findings point to the potential of using self-care skills, of employing a flexible tapering procedure, and of forming a good alliance between the patient and the prescribing clinician. The findings also point to the differential prediction of successful discontinuation of AP *v.* AD. For antipsychotic discontinuation, an individualized, cautious and well-prepared tapering strategy is most likely to be successful. For antidepressant discontinuation, a good therapeutic alliance seems crucial, whereas the role of the tapering strategy might be less straightforward, with this study pointing to benefits of an abrupt approach. However, these findings require further research that uses significantly larger samples to be able to differentiate between full and partial discontinuation and to take moderating factors into account. The complexity of the prediction might also be an indication for a precision medicine approach, using machine learning to optimize discontinuation strategy selection.

## Supplementary material

For supplementary material accompanying this paper visit https://doi.org/10.1017/S0033291721005146. click here to view supplementary material
