# Supplementary material for: Predictors of successful discontinuation of antipsychotics and antidepressants
Source: Psychol Med. 2021 Dec 23;53(7):3085–95. doi: 10.1017/S0033291721005146 (PMC10235642; doi:10.1017/S0033291721005146)
Supplement: Supplementary file 1 [file S0033291721005146sup.zip › S0033291721005146sup001.docx]

**Supplement 1 - Factor analysis results for predictor variables**

1. Discontinuation strategy
   1. Table S1
   2. Table S2
2. Mindful self care scale (MSCS)
   1. Table S3 - Loadings for EFA and CFA of the MSCS

**1. Discontinuation strategy**

The first part of the assessment (*preparation* and *tapering) included 7 seven items.* Exploratory factor analysis (EFA) with varimax rotation yielded 1 item with substantial cross-loading which was excluded (see Table S1, left). The remaining 6 items consisted of 3 items loading on the factor preparation and 3 items loading on the factor tapering. Confirmatory factor analysis (CFA) of this two factor model yielded h sufficient model fit (CFI=0.991, RMSEA=0.060, SRMR=0.030), with all items showing high loadings (0.634-0.952) to their respective factors.

Table S1-1.

Factor analysis of the first part of the discontinuation strategy assessment

|  | EFA | | CFA | |
| --- | --- | --- | --- | --- |
| Item content | Tapering | Preparation | Factor allocation | Standardized loading |
| I gathered information about discontinuation in advance | 0.338 | **0.583** | Preparation | 0.699 |
| I made preparations before starting to discontinue | 0.166 | **0.606** | Preparation | -0.759 |
| I didn’t gave it much thought before discontinuing | -0.231 | **-0.739** | Preparation | -0.632 |
| I discontinued my medication in a stepwise procedure | **0.905** | 0.284 | Tapering | 0.952 |
| I discontinued my medication all at once | **-0.878** | -0.270 | Tapering | -0.915 |
| I followed a detailed plan on how to reduce my medication | **0.682** | 0.398 | Tapering | 0.768 |
| I didn’t follow any discontinuation plan | -0.459 | -0.517 | - | - |

Note. The highest loading of an item with substantial difference from the other loading (>0.1) in EFA is printed in bold.

For the second part, participants indicating they were following a tapering plan (*n*=247) answered 4 items on *adjustment phases* and *flexible change*. Principal component analysis (PCA) yielded the two components with eigenvalues above 1 that had 2 items loading on them. Since a factor analysis with 4 items and two factors could not be calculated, the PCA loadings guided the item-allocation (see Table S2, left). Since a CFA with only 2 item factors prevents the calculation of model fit, only the loadings were used as an indicator in CFA (Table S2).

Table S1-2.

Results of PCA and CFA of adjustment phases and flexible change

|  | PCA | | CFA | |
| --- | --- | --- | --- | --- |
| Item | Comp 1 | Comp 2 | Factor allocation | Standardized loading |
| I only continued discontinuation when I felt stable enough with the current dosage. | **0.654** | -0.268 | Adjustment phases | 0.693 |
| I let sufficient time pass between reduction steps so I could get used to the new dosage. | **0.666** | -0.222 | Adjustment phases | 0.972 |
| I followed my gut feeling when discontinuing. | 0.197 | **-0.705** | Flexible reduction | 0.318 |
| I returned to a higher dosage when I had the feeling I reduced it too fast. | 0.299 | **0.618** | Flexible reduction | 0.775 |

Note. The highest loading of an item in PCA is printed in bold.

**2. Mindful Self Care Scale (MSCS)**

Since the MSCS was developed to assess interrelated aspects of self-care, EFA was calculated with a promax rotation. The loadings of a promax-rotated exploratory factor analysis are shown in Table S3. Three items for physical self-care showed were removed from the scale due to low loadings in the EFA and three items loaded on different factors and where added to this factor (see original allocation and CFA-allocation in Table S3). One item from the relaxation factor showed cross-correlation with the supportive structure factor, but was not omitted or changed due to sufficiently high loading on its original factor. CFA was calculated for the original item-set/-allocation (CFI=0.834, RMSEA=0.070, SRMR=0.081) and the revised item set and allocation (CFI=0.909, RMSEA=0.056, SRMR=0.063). With sufficient model fit only found in the revised factor model, the revised scale was used for all analysis in this article.

Table S1-3.

Factor loadings from EFA and CFA of the Mindful Self Care Scale (MSCS)

|  | EFA | | | | | | CFA | | | |
| --- | --- | --- | --- | --- | --- | --- | --- | --- | --- | --- |
| Item content | Supportive relationships | Supportive structures | Mindful awareness | Physical care | Compassion | Relaxation | Original allocation | revised allocation | Loadings (original) | Loadings (revised) |
| I drank at least 6 to 8 cups of water. |  | -0.176 |  | 0.167 |  | 0.211 | physical care | - | 0.177 |  |
| I ate a variety of nutritious foods (e.g., vegetables, protein, fruits, and grains). |  |  | 0.100 | **0.289** |  | 0.121 | physical care | | 0.353 | 0.324 |
| I planned my meals and snacks. |  | 0.321 |  | 0.168 |  |  | physical care | - | 0.308 |  |
| I exercised at least 30 to 60 minutes. |  | -0.124 |  | **0.888** |  |  | physical care | | 0.829 | 0.831 |
| I took part in sports, dance or other scheduled physical activities (e.g., sports teams, dance classes). | 0.130 |  |  | **0.429** | -0.187 |  | physical care | | 0.483 | 0.472 |
| I did sedentary activities instead of exercising (e.g., watched TV, worked on the computer). |  | -0.157 |  | **-0.601** |  | 0.137 | physical care | | -0.599 | -0.591 |
| I planned/scheduled my exercise for the day. |  |  |  | **0.904** | 0.105 |  | physical care | | 0.881 | 0.899 |
| I practiced yoga or another mind/body practice (e.g., Tae Kwon Do, Tai Chi) |  | 0.262 |  | 0.242 |  | 0.136 | physical care | - | 0.381 |  |
| I spent time with people who are good to me (e.g., support, encourage, and believe in me). | **0.772** |  |  |  |  |  | supportive relationships | | 0.790 | 0.803 |
| I scheduled/planned time to be with people who are special to me. | **0.884** | -0.111 |  |  |  |  | supportive relationships | | 0.867 | 0.850 |
| I felt supported by people in my life. | **0.863** |  |  |  |  |  | supportive relationships | | 0.778 | 0.763 |
| I felt confident that people in my life would respect my choice if I said “no”. | **0.531** | 0.149 |  |  |  | -0.187 | supportive relationships | | 0.553 | 0.548 |
| I felt that I had someone who would listen to me if I became upset (e.g., friend, counselor, group). | **0.626** | 0.129 |  |  | 0.112 | 0.109 | supportive relationships | | 0.777 | 0.789 |
| I had a calm awareness of my thoughts. |  |  | **1.043** |  |  |  | mindful awareness | | 0.927 | 0.926 |
| I had a calm awareness of my feelings. |  |  | **0.805** |  | 0.141 |  | mindful awareness | | 0.890 | 0.891 |
| I had a calm awareness of my body. |  |  | **0.862** |  |  |  | mindful awareness | | 0.879 | 0.878 |
| I carefully selected which of my thoughts and feelings I used to guide my actions. |  |  | **0.438** |  | 0.180 | 0.13 | mindful awareness | | 0.620 | 0.620 |
| I kindly acknowledged my own challenges and difficulties. |  |  | 0.136 |  | **0.662** |  | Compassion | | 0.703 | 0.749 |
| I engaged in supportive and comforting self-talk (e.g., “My effort is valuable and meaningful”). |  |  | -0.123 | 0.141 | **0.799** |  | Compassion | | 0.735 | 0.787 |
| I reminded myself that failure and challenge are part of the human experience. |  | 0.217 |  |  | **0.584** |  | Compassion | | 0.710 | 0.727 |
| I gave myself permission to feel my feelings (e.g., allowed myself to cry). |  |  |  |  | **0.579** | 0.182 | Compassion | | 0.549 | 0.584 |
| I experienced meaning and/or a larger purpose in my work/school life (e.g., for a cause). |  | **0.576** |  |  |  |  | Compassion | Supp. struct. | 0.477 | 0.566 |
| I experienced meaning and/or a larger purpose in my private/personal life (e.g., for a cause). | 0.319 | **0.602** | -0.113 |  |  |  | Compassion | Supp. struct. | 0.615 | 0.725 |
| I did something intellectual (using my mind) to help me relax (e.g., read a book, wrote). |  | 0.334 |  | -0.173 |  | 0.396 | Relaxation | | 0.494 | 0.569 |
| I did something interpersonal to relax (e.g., connected with friends). | **0.552** |  |  |  |  | 0.208 | Relaxation | Supp. relat. | 0.714 | 0.708 |
| I did something creative to relax (e.g., drew, played instrument, wrote creatively, sang, organized). |  | 0.106 |  |  | -0.110 | **0.427** | Relaxation | | 0.408 | 0.452 |
| I listened to relax (e.g., to music, a podcast, radio show, rainforest sounds). |  | -0.224 |  |  |  | **0.775** | Relaxation | | 0.424 | 0.530 |
| I sought out images to relax (e.g., art, film, window shopping, nature). |  |  |  |  |  | **0.582** | Relaxation | | 0.460 | 0.610 |
| I sought out smells to relax (lotions, nature, candles/incense, smells of baking). | -0.141 | 0.145 |  |  |  | **0.387** | Relaxation | | 0.299 | 0.409 |
| I maintained a manageable schedule. |  | **0.760** |  |  | -0.104 | -0.135 | Supportive structures | | 0.517 | 0.524 |
| I kept my work/schoolwork area organized to support my work/school tasks. |  | **0.727** |  |  | 0.155 | -0.175 | Supportive structures | | 0.644 | 0.619 |
| I maintained balance between the demands of others and what is important to me. |  | **0.612** |  | -0.107 | 0.278 |  | Supportive structures | | 0.749 | 0.702 |
| I maintained a comforting and pleasing living environment. |  | **0.624** |  |  |  | 0.152 | Supportive structures | | 0.734 | 0.731 |
| CFI |  |  |  |  |  |  |  |  | 0.834 | 0.909 |
| RMSEA |  |  |  |  |  |  |  |  | 0.070 | 0.056 |
| SRMR |  |  |  |  |  |  |  |  | 0.081 | 0.063 |

Note. The highest loading of an item with substantial difference from the other loading (>0.1) in EFA is printed in bold.
