# Supplementary material for: Predictors of successful discontinuation of antipsychotics and antidepressants
Source: Psychol Med. 2021 Dec 23;53(7):3085–95. doi: 10.1017/S0033291721005146 (PMC10235642; doi:10.1017/S0033291721005146)
Supplement: Supplementary file 1 [file S0033291721005146sup.zip › S0033291721005146sup002.docx]

**Supplement 2 – Subgroup control analysis for objective discontinuation of antidepressants (AD) and antipsychotics (AP)**

In our main analyses, the dependent variable *objective discontinuation* included the two groups:

1= *people who reported to no longer take the medication they intended to discontinue*

and

0= *people who had not reached that goal and were either taking the same, a higher or a lower dose of the medication they had set out to discontinue*.

The latter group consisted in large part of people who had reduced their dosage and included participants who had only recently started their discontinuation attempt and might therefore be on track towards full discontinuation. To rule out that the results of these analyses were unduly affected by this type of participants, we re-ran the analyses while factoring out the potential influence of people who had not yet achieved the full discontinuation status, but could still reasonably be expected to succeed.

In order to do this, we repeated the single predictor analyses with a subsample that excluded participants who met all of the following two conditions:

(1) they reported that their current discontinuation attempt was not finished,

(2) their current discontinuation attempt began less than a year ago, and

(3) they were still taking a reduced or the same amount of medication as they started with. This lead to the exclusion of 63 participants (of which 53 were currently taking a reduced dosage and 10 were still taking the starting dosage).

The following tables show a comparison of the single predictor regression analyses with the full sample vs the sample without ongoing attempts that recently started (AD sample: Table S2-1; AP: Table S2-2).

As can be seen, no previously significant effect changed direction in either the AD or the AP group (i.e., no predictor that was associated with an increased chance of objective discontinuation, OR<1, became a significant [or non-significant] predictor with OR>1,).

For AD discontinuation, all previously significant predictors remained significant in the subsample analyses and all non-significant effects remained non-significant. For AP, only the effect of gender remained significant. The effect of therapeutic alliance became a trend effect and the effect of unstable situation became non-significant with a comparable point-estimate of the OR. Finally, the flexible reduction effect became non-significant with a reduction of the point estimate of OR by descriptive values. Additionally, it needs noting that the sample of not successfully discontinuing participants became very small (*n*=22) in the AP subsample analysis, which might have affected test power.

Based on the consistent pattern of effects for AD, the consistency regarding descriptive values for AP, and bearing in mind the changes in test power that might lead to higher rates of non-significance we deemed the primary analysis to be sufficiently stable and continued the multiple regression analyses with the full samples.

Table S2-1.

Single predictor regression analyses for objective discontinuation by all putative psychosocial predictors in the antidepressant discontinuation group, full sample vs. subsample with an ongoing, recently started discontinuation attempt excluded.

|  | Objective discontinuation vs not (reduction/same dose/increase) | | | |
| --- | --- | --- | --- | --- |
|  | All participants | | Subsample, excluding those with an ongoing recently started discontinuation attempt | |
| Sample sizes | Successful: n=139  Not successful: n=128 | | Successful: n=139  Not successful: n=69 | |
| Predictor | OR | z | OR | z |
| Sociodemographic data |  |  |  |  |
| Age | 0.99 | -0.83 | 0.98 | -1.28 |
| Gender (female vs. male) | **0.43**** | **-2.84** | **0.40*** | **-2.47** |
| Education (high vs. low) | 1.19 | 0.66 | 0.94 | -0.19 |
| Clinical and medication history |  |  |  |  |
| Duration of disorder | 1.02 | -0.90 | 0.98 | -1.53 |
| Discontinuation of SSRI | 1.18 | 0.07 | 1.16 | 0.49 |
| Discontinuation of SNRI | 0.79 | -0.92 | 0.69 | -1.22 |
| Discontinuation of tri/tetracyclic AD | **3.08^**^** | **3.10** | **3.50**** | **2.66** |
| Duration of medication intake | 0.98 | -1.17 | 0.96^+^ | -1.89 |
| Multiple discontinued drugs (vs. one) | 1.37 | 1.11 | 1.24 | 0.63 |
| Concomitant medication | 0.61 | -1.44 | 0.61 | -1.44 |
| Professional support |  |  |  |  |
| Shared decision-making | 0.99 | -0.60 | 0.99 | -0.56 |
| Therapeutic alliance | 0.91 | -1.35 | 0.91 | -1.12 |
| Psychotherapy during attempt | 0.97 | -0.14 | 1.11 | 0.34 |
| Circumstances |  |  |  |  |
| Unstable situation | **1.03*** | **2.15** | **1.03*** | **2.04** |
| Discontinuation strategy |  |  |  |  |
| Preparation | 0.77^+^ | -1.89 | 0.73^+^ | -1.80 |
| Followed a tapering plan | **0.67***** | **-3.38** | **0.67**** | **-2.74** |
| Adjustment phases^1^ | **0.71**** | **-2.99** | **0.69*** | **-2.57** |
| Flexible reduction^1^ | 1.20 | 1.44 | 1.16 | 0.99 |
| Accelerated pacing (vs. slow)^1^ | **2.40*** | **2.17** | **2.61*** | **2.17** |
| Coping strategies |  |  |  |  |
| Physical care | 0.96 | -0.27 | 0.93 | -0.41 |
| Relaxation | 0.95 | -0.27 | 1.06 | 0.32 |
| Mindful awareness | 0.98 | -0.14 | 1.09 | 0.56 |
| Self-compassion | 1.12 | 0.85 | 1.08 | 0.51 |
| Supportive relationships | 0.78^+^ | -1.82 | 0.85 | -1.03 |
| Supportive structures | **0.71*** | **-2.40** | **0.70*** | **-2.09** |
| Drug consumption | 1.26 | 1.62 | 1.30 | 1.50 |

Note: Regression analyses with one predictor on the dependent variable objective discontinuation. ^1^Only participants who indicated to have followed a tapering plan responded to the questions on the details of the tapering plan.

Significance levels: + - p<0.1, * -p<0.05, ** -p<0.01, *** -p<0.001. Significant effects are printed in bold.

Table S2-2.

Single predictor regression analyses for objective discontinuation by all putative psychosocial predictors in the antipsychotic discontinuation group, full sample vs. subsample with an ongoing, recently started discontinuation attempt excluded.

|  | Objective discontinuation vs not (reduction/same dose/increase) | | | |
| --- | --- | --- | --- | --- |
|  | All participants | | Subsample, excluding those with an ongoing recently started discontinuation attempt | |
| Predictor | OR | z | OR | z |
| Sample sizes | Successful: n=52  Not successful: n=42 | | Successful: n=52  Not successful: n=22 | |
| Sociodemographic data |  |  |  |  |
| Age | 0.99 | -0.69 | 0.96^+^ | -1.91 |
| Gender (female vs. male) | **0.31*** | **-2.33** | **0.24*** | **-2.06** |
| Education (high vs. low) | 0.76 | 0.62 | 0.55 | -1.00 |
| Clinical and medication history |  |  |  |  |
| Duration of disorder | 1.00 | 0.14 | 0.97 | -1.10 |
| Discontinuation of 1^st^ gen. AP | 0.74 | -0.64 | 0.71 | -0.60 |
| Discontinuation of 2^nd^ gen. AP | 2.40 | 1.32 | 2.67 | 1.29 |
| Duration of medication intake | 0.99 | -0.26 | 0.96 | -1.15 |
| Multiple discontinued drugs (vs. one) | 0.57 | -1.33 | 0.64 | -0.86 |
| Concomitant medication | 0.76 | 0.54 | 1.07 | 0.11 |
| Professional support |  |  |  |  |
| Shared decision-making | 0.98 | -1.39 | 0.98 | -1.07 |
| Therapeutic alliance | **0.69**** | **-0.37** | 0.76^+^ | -1.83 |
| Psychotherapy during attempt | 0.79 | -0.55 | 0.97 | -0.06 |
| Circumstances |  |  |  |  |
| Unstable situation | **1.05*** | **1.97** | 1.04 | 1.50 |
| Discontinuation strategy |  |  |  |  |
| Preparation | 0.71 | -1.49 | 0.62 | -1.55 |
| Followed a tapering plan | 0.74 | -1.50 | 0.67 | -1.50 |
| Adjustment phases^1^ | 1.02 | 0.10 | 1.26 | 0.95 |
| Flexible reduction^1^ | **1.54*** | **2.03** | 1.11 | 0.44 |
| Accelerated pacing (vs. slow)^1^ | 0.51 | -1.07 | 0.55 | -0.81 |
| Coping strategies |  |  |  |  |
| Physical care | 0.81 | -0.91 | 0.66 | -1.42 |
| Relaxation | 1.10 | 0.33 | 0.97 | -0.10 |
| Mindful awareness | 0.89 | -0.59 | 0.85 | -0.60 |
| Self-compassion | 0.98 | -0.07 | 0.67 | -1.30 |
| Supportive relationships | 0.77 | -1.13 | 0.79 | -0.81 |
| Supportive structures | 0.84 | -0.71 | 0.62 | -1.52 |
| Drug consumption | 1.52^+^ | 1.90 | 1.36 | 1.20 |

Note: Regression analyses with one predictor on the dependent variable objective discontinuation. ^1^Only participants who indicated to have followed a tapering plan responded to the questions on the details of the tapering plan.

Significance levels: + - p<0.1, * -p<0.05, ** -p<0.01, *** -p<0.001. Significant effects are printed in bold.
